# From Sanger to Oxford Nanopore MinION Technology: The Impact of Third-Generation Sequencing on Genetic Hematological Diagnosis

**DOI:** 10.3390/cancers17111811

**Published:** 2025-05-29

**Authors:** María José Larráyoz, Pablo Luri-Martin, Amagoia Mañu, Oihane Churruca, Natalia Gordillo, Irache Erdozain, Ada Esteban-Figuerola, Carlos de Miguel, Diego Robles, María García-Fortes, José Rifón Roca, Ana Alfonso-Pierola, Felipe Prósper, Beñat Ariceta, María José Calasanz

**Affiliations:** 1 Hematological Diseases Laboratory, CIMA LAB Diagnostics, Clinica Universidad de Navarra, 31008 Pamplona, Spain; mjlarra@unav.es (M.J.L.); pluri@unav.es (P.L.-M.);; 2 Navarra Institute for Health Research (IdiSNA), 31008 Pamplona, Spain; 3 Traslational Hematology Program, Center for Applied Medical Research (CIMA), Cancer Center University of Navarra (CCUN), 31008 Pamplona, Spain; 4 Special Hematology Laboratory, Hematology Department, San Pedro Hospital, 26006 Logroño, Spain; 5 Department of Hematology, Hospital Universitario de Álava—Sede Txagorritxu, 01009 Vitoria-Gasteiz, Spain; 6 Hematology Department, Hospital Universitario Virgen de la Victoria, 29010 Málaga, Spain; 7 Hematology Department, Clinica Universidad de Navarra (CUN), Cancer Center University of Navarra (CCUN), 31008 Pamplona, Spain; 8 CIBERONC, 28029 Madrid, Spain

**Keywords:** MinION technology, Oxford Nanopore MinION device, Sanger sequencing, oncohematology, mutational analysis, myeloproliferative neoplasms, acute myeloid leukemia, myelodysplastic syndromes, chronic myeloid leukemia, B-cell chronic lymphocytic leukemia

## Abstract

Background: Sanger sequencing remains the gold standard for characterizing genetic variants in short DNA fragments (<700 bp). However, the increasing demand for short TATs and high sensitivities in variant detection, particularly in oncohematology, is driving the need for more efficient methods. Next-generation sequencing (NGS) has improved sensitivity and allows for the simultaneous analysis of multiple genes, but it is still costly and time-consuming. Consequently, Sanger sequencing continues to be widely used. In this study, we have compared Sanger sequencing with Oxford Nanopore technology (ONT), which offers enhanced sensitivity and faster sequencing, delivering diagnostic results within 24 h. Methods: This study involves 164 samples (for a total of 174 analyzed regions of interest) previously characterized using either Sanger sequencing or a next-generation sequencing (NGS) panel, categorized by their genetic alterations. Validation was conducted on 15 genes crucial for the diagnosis, prognosis, or identification of drug resistance in myeloproliferative neoplasms (MPN), myelodysplastic syndromes (MDS), acute myeloid leukemia (AML), and chronic myeloid leukemia (CML). The primary objective was to assess whether MinION could identify the same variants previously detected in these patients. Results and Conclusions: With a 99.43% concordance observed in our comparison, our results support the implementation of MinION technology in routine variant detection in MPN, MDS, AML, and CML cases due to its significant advantages over Sanger sequencing.

## Simple Summary

With the aim of advancing and innovating improvements in genetic diagnosis, this work aims to compare traditional Sanger sequencing with MinION technology for detecting variants in short fragments. The routine use of this third-generation technology is expected to have a tremendous positive impact on improving the turnaround time (TAT), among other factors, directly contributing to improvements in patients’ health.

## 1. Introduction

Sanger sequencing remains crucial for detecting single-nucleotide variants and small insertions/deletions (INDELs) in hematological malignancies. It is routinely employed in molecular diagnostic laboratories for sequencing individual genes and small targeted genome regions. NGS has not fully replaced Sanger sequencing because, for conditions resulting from mutations in a single gene or a small group of genes, it is impractical to analyze the large number of genes included in an NGS panel. Moreover, there are cases of genes (such as *FLT3*, *IDH1*, and *NPM1*) that, due to treatment or inclusion in a trial, require a short TAT, which is currently not offered by NGS but is achievable with Sanger sequencing. On the other hand, MinION can offer the same or greater speed than Sanger in these cases, but also with a sensitivity like that of NGS. Hence, one of the major interests is replacing Sanger sequencing with ONT technology.

Returning to the applications that Sanger sequencing still has today in genetic diagnostics laboratories, nowadays, performing mutational analysis on a single gene or a few relevant genes using Sanger is more practical, faster, and cost-effective than with NGS. In some situations, we examine the entire coding region, such as the *CEBPA* gene. In other cases, we target specific exons where the main hotspots of that gene are located, such as with the *KIT* gene, in which, in routine diagnostics, we study exon 17 (p.D816V) and exon 8 because they contain variants that provide a definite diagnosis of mastocytosis [1,2] and are associated with an unfavorable prognosis in AML patients [3,4]. Regarding the previously mentioned example highlighting the continued importance of Sanger sequencing in cases requiring a very short turnaround time (TAT), the case of the *NPM1* gene is particularly noteworthy. *NPM1* is a favorable prognostic marker in newly diagnosed AML [5,6,7], and thus, timely delivery of its mutational status to the clinician is critical to support the most appropriate therapeutic decision. Obtaining such rapid results with NGS techniques is currently not feasible.

Current routine molecular gold standard methods include Sanger and NGS. In this study, we aim to validate ONT as a new approach for the molecular characterization of hematological malignancies. Specific characteristics of each method are described in the following table (Table 1).

Since its launch in 2014 by Oxford Nanopore Technologies (ONT), nanopore technology has been widely adopted for sequencing large fragments, including viral, bacterial, and yeast genomes [10,11,12]. For instance, the MinION was used in Africa for screening Ebola and Lassa virus outbreaks [13,14]. However, it has been less commonly used for sequencing short fragments and is even rarer in oncohematological diagnosis. The most notable studies utilizing MinION in oncohematology have focused on detecting mutations in the *TP53* [15] and the *ABL1* [16] genes in B-cell chronic lymphocytic leukemia (B-CLL) and CML patients, respectively. Additionally, a customized MinION-based gene panel has been developed for the targeted sequencing of genes frequently mutated in B-CLL [17]. More recently, a nanopore-based assay has been introduced for the rapid sequencing of six genes frequently mutated in AML (*NPM1*, *FLT3*, *CEBPA*, *TP53*, *IDH1*, and *IDH2*) [18].

Herein, as we explore the potential of MinION technology as a new approach in the clinical routine for short-fragment sequencing, we used a substantial cohort of 164 samples, previously characterized using NGS or Sanger sequencing, targeting 15 genes with diagnostic, prognostic, or therapeutic relevance in MPN (*CALR*, *JAK2*, *MPL*, *CSF3R*, *SETBP1)*, MDS (*SF3B1)*, AML (*NPM1*, *KIT*, *IDH2*, *IDH1*, *CEBPA*, *NRAS*, *KRAS*, and *TP53)* and CML (*ABL1*). This approach seeks to evaluate MinION’s feasibility and performance in the context of oncohematological diagnostics.

## 2. Materials and Methods

**Figure 1:**
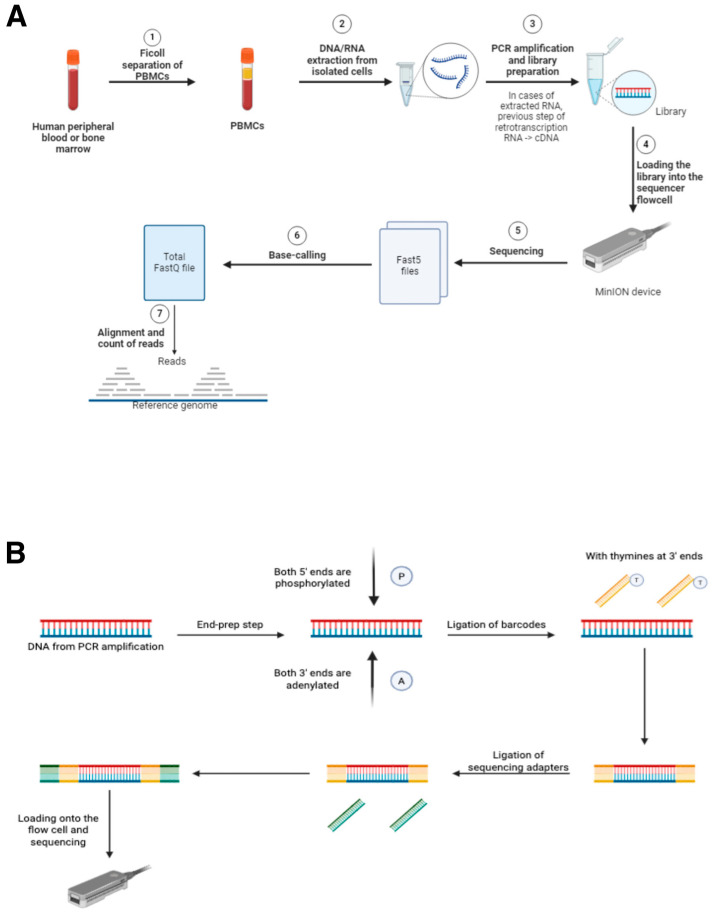
(**A**) Workflow for the use of MinION as a diagnostic tool in oncohematology. Steps 5, 6, and 7 require the use of MinKNOW software and are necessary for sequencing analyses. (**B**) Schematic figure showing the library preparation process for sequencing with MinION. The use of barcodes is necessary in the case of multiplexing, where multiple samples will be sequenced at the same time in a single sequencing run. All the reagents required for these steps are provided by ONT in their sequencing kits. (Figure 1 has been created manually using the BioRender application (“https://www.biorender.com/”. Accesed date 23 May 2025).

The analysis workflow for each sample began with the receipt of human peripheral blood or bone marrow samples in our laboratories. Subsequently, peripheral blood mononuclear cells (PBMCs) were isolated using Ficoll separation (Cytiva Ficoll-Paque™ PREMIUM, Cytiva Life Sciences™, Thermo Fisher Scientific; Waltham, MA, USA). RNA or DNA, depending on the specific detection test for each marker, was extracted from the isolated cells. Marker-specific PCRs were subsequently conducted in accordance with the standard procedures established at the CIMA LAB Diagnostics laboratory (no marker-specific PCRs have been designed for these validations). Finally, the library was prepared according to the protocols provided by ONT, followed by loading the library onto the sequencer. Upon completion of sequencing, base-calling of the corresponding Fasta5 files was conducted, followed by alignment and read counting. This process is summarized in the following figure (Figure 1).

All this experimental process will be detailed below in the corresponding subsections of the Section 2.

### 2.1. Patients

One hundred and sixty-four patients were included in this study, categorized based on their diagnosed pathologies as follows: 58 MPN, 12 MDS, 84 AML, 9 CML, and 1 ALL (Table S1). The ALL patient served as a non-mutated control in the *ABL1* assay. Regarding the samples, a total of 101 out of 164 were bone marrow, while 63 out of 164 were peripheral blood.

The samples used for the validations were collected between 2016 and 2024. All patients provided written informed consent for genetic testing, research, and tissue banking through the Biobank of the University of Navarra. Sample processing adhered to the standard operating procedures approved by the Institutional Review Board (IRB) of the University of Navarra.

The design of each assay utilized samples that had been previously characterized in the molecular biology laboratory at CIMA LAB Diagnostics, using either Sanger sequencing or an NGS panel. Leveraging samples characterized by an NGS panel for the validation of MinION technology offers a distinct advantage, as it enables the detection of variants that may not be identified by Sanger sequencing due to its lower sensitivity (note that NGS sensitivity can be as high as 1%).

The project was structured into individual assays per gene, each corresponding to a specific pool loaded onto the sequencer. For each pool, between 11 and 13 samples of each gene were analyzed, comprising six to eight samples with known variants and four to five control samples without variants. An exception was made for the *NPM1* gene, where the initial assay design included a total of 10 samples with mutations and two control samples without mutations. For cases with mutations, only the exon containing the variant(s) was amplified, whereas in non-mutated cases, all exons routinely sequenced were amplified.

### 2.2. Polymerase Chain Reaction

The samples selected for each assay comprised stored DNA or RNA (either in the Biobank of our center or in our laboratory) previously extracted from mononuclear cell isolates obtained by Ficoll (Cytiva Ficoll-Paque™ PREMIUM, Cytiva Life Sciences™, Thermo Fisher Scientific; Waltham, MA, USA) from the bone marrow (BM) or peripheral blood (PB) samples collected at diagnosis for variant detection. DNA extraction was performed using the QIAamp DNA Mini Kit from QIAGEN (Hilden, Germany), while RNA was extracted using the RNeasy Mini Kit (250) from QIAGEN (Hilden, Germany). Quantification was performed using the Nanodrop (ND-1000) from Thermo Fisher Scientific (Waltham, MA, USA).

As previously noted, for the mutated samples, only the exon containing the variant was amplified. In the non-mutated control samples, all exons routinely sequenced in our laboratories were amplified. The sequence of the primers used for each gene under study is detailed in the Supplementary Material (Table S2). All primers were synthesized by Invitrogen (Thermo Fisher Scientific; Waltham, MA, USA), except for those targeting *ABL1*, which were synthesized by Sigma-Aldrich (St. Louis, MO, USA). Primer design was based on previously published studies or generated using the Primer3Plus software (version: 3.3.0). These specifications, as mentioned, are provided in the Supplementary Material (Table S2).

The common reagents used in our PCRs included the following: Braun nuclease-free water, 25 mM MgCl_2_ solution from Applied Biosystems (Thermo Fisher Scientific; Waltham, MA, USA), 10x PCR Gold Buffer from Applied Biosystems (Thermo Fisher Scientific; Waltham, MA, USA), dNTPs from Invitrogen (Thermo Fisher Scientific; Waltham, MA, USA), and AmpliTaq Gold^TM^ DNA Polymerase 250U from Applied Biosystems (Thermo Fisher Scientific; Waltham, MA, USA). For the *CEBPA* and *ABL1* genes, the enzyme used was Platinum^TM^Taq DNA Polymerase High Fidelity from Invitrogen (Thermo Fisher Scientific; Waltham, MA, USA); the 10x PCR Gold Buffer was also replaced by 10x High Fidelity PCR Buffer from Invitrogen (Thermo Fisher Scientific; Waltham, MA, USA). In addition, DMSO from Sigma-Aldrich (St. Louis, MO, USA) was included in the PCR mix to accommodate the high GC content of the *CEBPA* gene. Furthermore, for *TP53* exon 7 and *SF3B1* exons 13–14, the 10x PCR Gold Buffer was replaced by the AmpliTaq Gold DNA Polymerase Buffer II from Applied Biosystems (Thermo Fisher Scientific; Waltham, MA, USA).

The PCR results were visualized using capillary electrophoresis with the QIAxcel instrument from QIAGEN (Hilden, Germany). The PCR products were then purified with ExoSAP-IT™ PCR Product Cleanup Reagent from Thermo Fisher Scientific (Waltham, MA, USA). Before beginning library preparation, the products were quantified, and their purity was estimated using the Nanodrop ND-1000 (Thermo Fisher Scientific; Waltham, MA, USA).

### 2.3. Sanger Sequencing and NGS Panel

As previously mentioned, the molecular characterization of the samples used in the earlier validations was performed using Sanger sequencing or an NGS panel. The Sanger sequencing was conducted at the Madrid facility of Macrogen, INC (Seoul, Republic of Korea). The NGS panel was custom designed by CIMA LAB Diagnostics in collaboration with the University Hospital of Salamanca and manufactured by SOPHiA GENETICS (Saint-Sulpice, Switzerland): “Custom Panmyeloid Panel v2”. This panel simultaneously analyzes a total of 56 genes (complete/regions): *ACD*, *ANKRD26*, *ASXL1*, *ATRX*, *BCOR*, *BCORL1*, *CALR*, *CBL*, *CEBPA*, *CSF3R*, *CSNK1A1*, *CUX1*, *DDX41*, *DHX34*, *DNMT3A*, *ETNK1*, *ETV6*, *EZH2*, *FLT3 (ex.11–20)*, *GATA1*, *GATA2*, *IDH1*, *IDH2*, *IKZF1*, *JAK2*, *KIT*, *KMT2A*, *KRAS*, *MBD4*, *MECOM*, *MPL*, *NF1*, *NPM1*, *NRAS*, *PHF6*, *PPM1D*, *PTPN11*, *RAD21*, *RUNX1*, *SAMD9*, *SAMD9L*, *SETBP1*, *SF3B1*, *SH2B3/LNK*, *SMC1A*, *SMC3*, *SRP72*, *SRSF2*, *STAG2*, *TERC*, *TERT*, *TET2*, *TP53*, *U2AF1*, *WT1*, and *ZRSR2*.

The results obtained from these sequencing efforts were stored in our company’s database (FileMaker Pro INC; Cupertino, CA, USA; version: 20.3.1.31 (10132023). It is also noteworthy that, as part of our company’s quality control assurance (QCA), we utilized a Laboratory Information Management System (LIMS) at the care level called Gestlab (Clinisys; Tucson, AZ, USA; version: 2024.2.8).

### 2.4. MinION Sequencing

According to the Native Barcoding Kit 24 V14 (SQK-NBD114.24) protocol by Oxford Nanopore Technologies (Oxford, UK), 200 fmol (130 ng for 1 kb amplicons) of PCR product per sample was end-prepared using the NEBNext Ultra II End Repair/dA-Tailing Module (New England Biolabs Inc; Ipswich, MA, USA). Subsequently, the Blunt/TA Ligase Master Mix (New England Biolabs Inc; Ipswich, MA, USA) was used to ligate the nanopore-specific Native Barcodes (NB01–NB24) to the DNA. Equimolar amounts of each barcoded amplicon were then pooled (using the Quick Ligation module from New England Biolabs Inc; Ipswich, MA, USA) and loaded into the MinION device (MinION Mk1B) through the Flow Cell (FLO-MIN114-R10.4.1). The sequencing parameters, device status, real-time sequencing, and other functions were managed using the ONT software MinKNOW, which was installed on a computer connected to the MinION via a USB port.

ONT also provides a protocol for washing and storing the sequencer’s Flow Cell (Flow Cell Wash Kit EXP-WSH004 & EXP-WSH004-XL).

### 2.5. MinION Sequencing Data Analysis

According to the manufacturer’s instructions, demultiplexing was performed during sequencing based on the nanopore-specific Native Barcodes with the automatic detection of present barcodes. The generated FASTQ files were transferred to in-house servers for downstream analysis in a High-Performance Computing cluster (HPC).

In summary, the NextFlow nanoseq [19] pipeline was used for downstream analysis. Alignment was conducted using minimap2 against the hg19 reference genome from demultiplexed FASTQ files, and only SNV detection was activated with a pepper-margin-deep variant caller. The resulting VCF file was processed to obtain only variants above the 5% threshold (selecting only positions with at least a 10,000X sequencing depth). Selected variants were annotated using the VEP Web Interface [20].

For the sensitivity analysis, only the samples specifically selected for this purpose were included. The analysis was conducted following the same procedure, but the final selection step was omitted to allow the annotation of the complete list of detected variants.

For further information, scripts used for MinION data analysis are published on GitHub (https://github.com/bariceta/MinAMP. Accessed date: 23 May 2025).

### 2.6. Statistical Analysis

To obtain the final statistical results, R (version 4.2.1) was utilized. The analysis was divided into a comparison of continuous data (NGS panel vs. ONT) and discrete results (Sanger vs. ONT). For discrete results, a confusion matrix was generated to calculate key performance metrics, and a binomial test was used for a *p*-value determination. For continuous VAF values, a correlation plot was created to compare the VAF values: the ground truth (VAF from the NGS panel) and the VAF obtained from the interrogated technology. Pearson’s correlation (R^2^) was calculated to evaluate the correlation between the two methods. The R packages used for the data analysis and graphical representation were yardstick, smplot2, caret, openxlsx, and ggplot2.

## 3. Results

On average, each sequencing run generated 3.23 million reads, yielding a mean of 1.83 Gb of estimated bases (1 Gb = one billion base pairs). Additionally, an average of 89% of the bases successfully passed the base-calling process, demonstrating efficient read quality. The N50 statistic, a key metric for sequencing quality, was confirmed to be accurate across all validations. This ensured that the sequencing quality was aligned with the average length of the amplicons sequenced in each run. The N50 values consistently met the required standards, confirming the reliability of the sequencing data.

**Figure 2:**
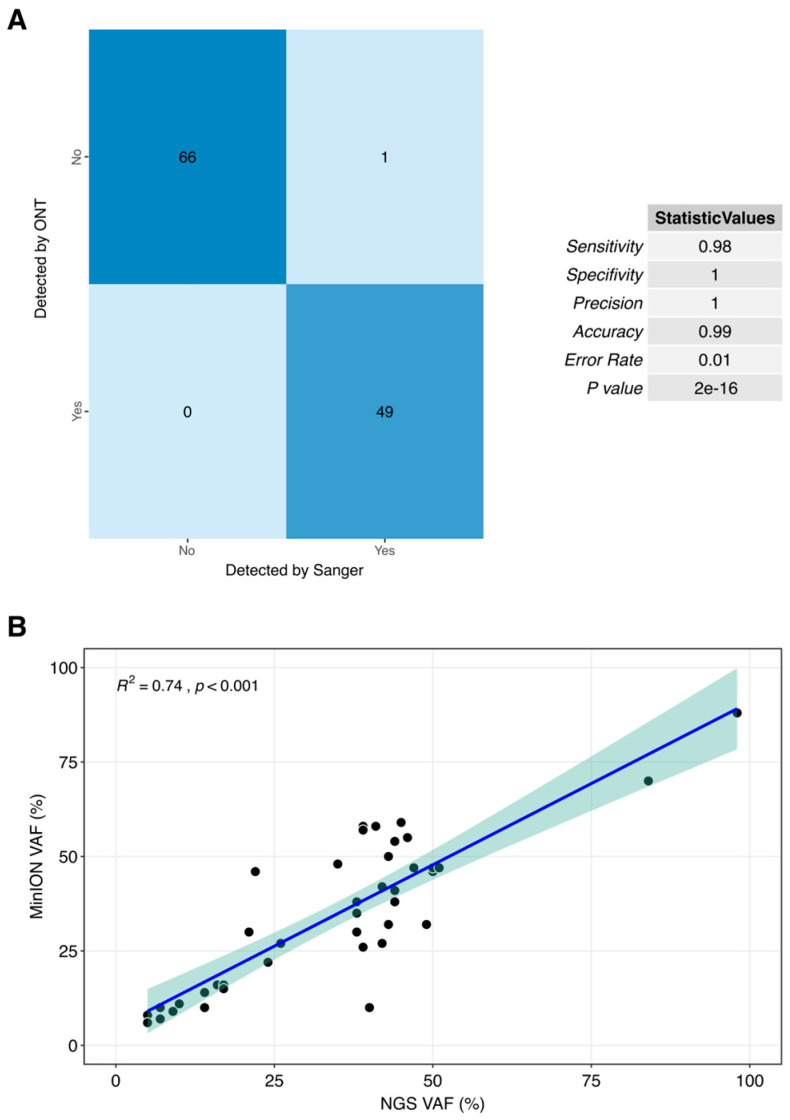
Correlation plots between current gold standard techniques, Sanger (**A**) and NGS targeted panel (**B**) and our proposed method for clinical routine. Each point refering to a unique sample with VAF values using both technologies with a 5% of deviance shadow.

Figure 2 highlighted the strong correlation observed between the results obtained using MinION and those generated by Sanger-NGS. This demonstrates the high level of agreement between the two technologies in our analysis, underscoring the reliability and accuracy of MinION in comparison with the established methods. Out of the 174 analyzed sites (comprising the total regions of interest across the 164 samples under study), only one discordant result was observed, reflecting a concordance rate of 99.43% (173/174) between the gold standard technique and the proposed method. When comparing the presence or absence of specific variants between Sanger and MinION-ONT, the statistical analysis demonstrated high accuracy, sensitivity, and specificity, all supported by a significant *p*-value (Figure 2A). In the comparison of VAF values, a strong correlation was observed between the different approaches, demonstrating that the differences between short-reads and long-reads are not significant in VAF detection (Figure 2B).

To present the results of the detected variants reported in Figure 2, the genes have been grouped based on their diagnostic and prognostic significance within specific pathologies. Consequently, a total of five tables (including one summarizing the sensitivity test results) correspond to the following pathologies: MPN, MDS, AML, and CML. Each of the five tables specifies the patient identifier, denoted by the hash symbol.

### 3.1. MPN

The MPN cases analyzed include samples from the following genes: *CALR*, *JAK2*, *MPL*, *CSF3R*, and *SETBP1*. In summary, among a total of 59 MPN regions of interest (ROI), the ONT demonstrated a 100% concordance. Detailed information for the MPN cases is presented in Table 2.

### 3.2. MDS

The MDS cases analyzed include samples from the *SF3B1* gene. In summary, among a total of 10 MDS ROI, a concordance with the ONT of 100% was seen. This information corresponding to the MDS cases is presented in Table 3.

### 3.3. AML

The AML cases analyzed include samples from the following genes: *NPM1*, *KIT*, *IDH2*, *IDH1*, *CEBPA*, *NRAS*, and *KRAS*. Among a total of 79 AML ROI, the ONT achieved a concordance of 98.73%, with only one discordant result identified among the 79 interrogated regions. The discordant case corresponds to a region of the *CEBPA* gene. It is important to note that this gene contains regions highly enriched in GC content, which makes it challenging to sequence and increases the likelihood of sequencing errors. It should also be considered that it was a variant validated by Sanger sequencing, and so the result of the sequencing could have been incorrect, falsely detecting a sequence alteration, whereas the ONT sequencing may, in fact, be accurate. The limitations of Sanger sequencing, particularly in GC-rich regions, must also be taken into consideration. This information corresponding to the AML cases is presented in Table 4.

### 3.4. CML

The CML cases analyzed include nine CML patients and one ALL patient as a negative control (non-mutated). These CML/ALL cases include samples from the gene *ABL1.* In summary, out of a total of 10 CML-ALL ROI, a concordance with the ONT of 100% was seen. This information is presented in Table 5.

### 3.5. Sensitivity Testing

Having demonstrated the efficacy of ONT for variant detection in hematological diagnosis and follow-up, showing that at VAFs > 5%, MinION can detect the same variants observed with the current gold standard (Sanger) or NGS technology, we aimed to further explore that nanopore sequencing not only matches the depth of NGS but also significantly surpasses the depth achievable with Sanger sequencing. To this end, we selected samples that had variants detected by NGS and whose VAFs were less than 5%. From a technical standpoint, achieving this level of depth required modifying the aforementioned analysis filter that retained only variants with a VAF greater than 5%. By making a simple adjustment to the analysis workflow, we were able to detect subclonal variants. These samples (13 in total, comprising 16 variants) are detailed in Table 6. As shown, all variants were successfully detected using both methods, further demonstrating the sensitivity and specificity of the proposed assay.

## 4. Discussion

Third-generation genomic nanopore technology (MinION) has revolutionized DNA sequencing and, consequently, the genetic diagnosis of hematological malignancies [21,22]. Although the advent of NGS represented a significant paradigm shift in genetic screening [23,24,25,26,27,28], nanopore sequencing developed by ONT is rapidly closing the gap. With its rapid sequencing speed, versatility (enabling the sequencing of both short fragments and entire genomes), compact design, and competitive price, nanopore technology stands at the forefront of innovation in DNA sequencing [29,30,31,32].

In the field of oncohematological diagnosis, nanopore sequencing represents a major paradigm shift. With the increasing focus on precision medicine, TAT has become a critical factor [33]. Rapidly identifying and administering the appropriate treatment is paramount for improving patient outcomes. MinION technology can deliver molecular results within 24 h [34] due to its real-time sequencing capabilities, offering a sensitivity comparable to NGS (<1%) and significantly surpassing Sanger sequencing (15%). This advancement not only enhances the TAT of Sanger sequencing (3–4 days in our laboratories at CIMA LAB Diagnostics) but also dramatically improves the TAT of NGS (approximately 14 days in our laboratories). This is arguably one of the strongest arguments in favor of implementing MinION technology, as the growing availability of personalized treatments makes rapid clinical decision-making increasingly critical, especially in acute diseases, where early and accurate therapeutic guidance can significantly influence patient prognosis.

Another significant advantage highlighted in this study is the remarkable efficiency of nanopore technology in detecting variants with allele frequencies below 5%. This capability represents a substantial improvement over Sanger sequencing. The ability to identify somatic variants in subpopulations of tumor cells, thus present in a low proportion in the tumor sample, is critically important in addressing relapse or treatment resistance in certain hematological tumors. This enhanced sensitivity can play a pivotal role in tailoring therapies and improving patient outcomes in oncohematology.

On a separate note, while MinION technology, as demonstrated in this study, excels in detecting variants in complex sequences such as those of the *CEBPA* gene, surpassing NGS in this specific aspect, it is not feasible to consider nanopore sequencing as a replacement for NGS. The capacity of next-generation sequencing to simultaneously analyze a broad spectrum of genes remains unmatched by MinION at this stage. However, as demonstrated in this study, it is both practical and advantageous to begin phasing out Sanger sequencing in favor of MinION for routine diagnostic applications. Therefore, MinION is affordable, efficient, and can be seamlessly integrated into molecular biology laboratories, enabling faster and more precise diagnostics.

Indeed, this pioneering approach is already being implemented in our molecular biology laboratory at CIMA LAB Diagnostics. We have initiated the routine use of MinION technology for detecting variants in the following 15 genes tested in this study: *CALR*, *JAK2*, *MPL*, *CSF3R*, *SETBP1*, *SF3B1*, *NPM1*, *KIT*, *IDH1*, *IDH2*, *CEBPA*, *NRAS*, *KRAS*, *TP53*, and *ABL1.*

The strong concordance observed in our analysis (99.43% (173/174)), which included a substantial cohort of patients and genes, strongly underscores the promising potential of nanopore sequencing for routine diagnostic use. Additionally, the exceptional sensitivity demonstrated by MinION in detecting variants (Table 6) highlights the significant utility and reliability of this technology in advancing the molecular diagnosis of hematological malignancies.

Beyond its demonstrated utility in oncohematology, nanopore sequencing technology showcases remarkable versatility due to its suitability for long-read sequencing, positioning it for numerous applications in genetic diagnostic laboratories. For instance, it has the potential to enhance classical cytogenetic techniques by accurately detecting translocations that are difficult to identify through conventional karyotyping, such as cryptic translocations or undescribed rearrangements. Such features represent a significant advantage of ONT, even over NGS. Long-read sequencing might lead to substantial improvements in the detection of copy number alterations (CNA) as well as gene fusions. This further highlights the remarkable potential impact that, beyond the findings demonstrated in this study, the implementation of ONT could have in genetic diagnostic laboratories.

## 5. Conclusions

This study highlights the effectiveness of MinION technology in detecting 174 regions of interest across 15 genes in a cohort of 164 samples. By validating the results against the current gold standards, both Sanger sequencing and NGS, this research confirms the applicability of MinION for molecular characterization in hematological diseases, including MPN, MDS, AML, and CML. These findings underscore the potential of MinION technology in comparison with Sanger sequencing for identifying genetic variants in short DNA fragments, offering a reliable, efficient, and cost-effective alternative. Moreover, MinION’s ability to deliver rapid results, even in real time, presents a significant advancement in diagnostic workflows, expediting critical clinical decisions. Its adoption could greatly benefit hematological patients and clinicians by facilitating timely and precise treatment selection, marking a crucial step forward in personalized medicine.

## Figures and Tables

**Table 1:** A comparison between Sanger sequencing, NGS, and MinION technologies.

	Sanger	NGS	MinION
**Sequencing method**	Termination by didesoxynucleotides	Massively parallel sequencing	Nanopore sequencing
**Single-read accuracy**	>99%	>99%	>99%
**Reading length**	400–900 base pairs	50–500 base pairs	Up to a megabase
**Time per run**	20 min–3 h	48 h *	1 min–48 h **
**Sensitivity**	15–20%	1%	<1% ***
**Error rate**	0.001%	0.1–1%	5% **** [8]
**Versatility**	Short-strand sequencing	Short-strand sequencing	Short- and long-strand sequencing
**Applications**	Single-nucleotide variants (SNVs) and INDELs detection	SNVs and INDELs Detection *	SNVs, INDELs, and complex structures detection (translocations, GC-rich regions, repetitive regions) [8,9]
**Turnaround time (TAT)**	3–4 days *****	Around 14 days *****	2–3 days *****, but real-time process, so in urgent cases, it could be under 24 h.
**Coverage**	A single read per sample	Often > 500X	Depending on the specific application (this case > 10,000X).

* Considering the sequencer (Illumina MiSeq) and the NGS panel (specified in the Section 2) used in our center: CIMA LAB Diagnostics. ** The process is in real time, so the results are visible from the first minute of sequencing. *** The analysis with MinION allows for obtaining a variant allele frequency (VAF) value equivalent to that offered by the NGS panels. This represents a significant advantage over Sanger sequencing, as it offers insights into the mutation frequency within cell populations. **** This is an area of ongoing improvement. Oxford Nanopore Technologies (ONT) continually updates its analysis software (MinKNOW 24.06) to reduce the error rate. In this study, two filters were applied during the analysis phase to mitigate the impact of this high error rate. These filters will be explained in detail later. ***** Considering the current setup of our laboratories at CIMA LAB Diagnostics. We outsource Sanger sequencing (as specified in the Section 2), which results in an extended turnaround time.

**Table 2:** Comparison of variants detected by the Sanger/NGS method vs. MinION. The genes included in this table have clinical significance in the diagnosis, prognosis, or development of MPN.

Gene	Sample ID	Gold Standard Method	Sanger/NGS Detected Variants [VAF Value]	MinION Detected Variants [VAF Value]	Matching Results
*CALR* exon 9	#1	Sanger	c.1154_1155insTTGTC; p.(K385Nfs*47)	c.1154_1155insTTGTC; p.(K385Nfs*47) [20%]	YES
	#2	Sanger	c.1154_1155insTTGTC; p.(K385Nfs*47)	c.1154_1155insTTGTC; p.(K385Nfs*47) [21%]	YES
	#3	Sanger	c.1099_1150del; p.(L367Tfs*46)	c.1099_1150del; p.(L367Tfs*46) [29%]	YES
	#4	NGS	c.1105_1138del; p.(E369Rfs*50) [39%]	c.1105_1138del; p.(E369Rfs*50) [58%]	YES
	#5	Sanger	c.1200_1220del; p.(D400_E406del)	c.1200_1220del; p.(D400_E406del) [34%]	YES
	#6	Sanger	c.1139A>G; p.(E380G)	c.1139A>G; p.(E380G) [46%]	YES
	#7	Sanger	Non-mutated	Non-mutated	YES
	#8	Sanger	Non-mutated	Non-mutated	YES
	#9	Sanger	Non-mutated	Non-mutated	YES
	#10	Sanger	Non-mutated	Non-mutated	YES
	#11	Sanger	Non-mutated	Non-mutated	YES
*JAK2* exon 14	#12	Sanger	c.1849G>T; p.(V617F)	c.1849G>T; p.(V617F) [21%]	YES
	#13	Sanger	c.1849G>T; p.(V617F)	c.1849G>T; p.(V617F) [36%]	YES
	#14	Sanger	c.1849G>T; p.(V617F)	c.1849G>T; p.(V617F) [7%]	YES
*JAK2* exon 12	#15	NGS	c.1612_1616delinsTT; p.(H538_K539delinsL) [7%]	c.1612_1616delinsTT; p.(H538_K539delinsL) [10%]	YES
	#16	Sanger	c.1623_1628delAAATGA; p.(N542_E543del)	c.1623_1628delAAATGA; p.(N542_E543del) [40%]	YES
	#17	Sanger	c.1627_1632delGAAGAT; p.(E543_D544del)	c.1627_1632delGAAGAT; p.(E543_D544del) [28%]	YES
*JAK2* exons 12 and 14	#18	Sanger	Non-mutated	Non-mutated	YES
	#19	Sanger	Non-mutated	Non-mutated	YES
	#20	Sanger	Non-mutated	Non-mutated	YES
	#21	Sanger	Non-mutated	Non-mutated	YES
	#22	Sanger	Non-mutated	Non-mutated	YES
*MPL* exon 10	#23	Sanger	c.1544G>T; p.(W515L)	c.1544G>T; p.(W515L) [37%]	YES
	#24	Sanger	c.1544G>T; p.(W515L)	c.1544G>T; p.(W515L) [41%]	YES
	#25	Sanger	c.1544G>T; p.(W515L)	c.1544G>T; p.(W515L) [70%]	YES
	#26	Sanger	c.1544G>T; p.(W515L)	c.1544G>T; p.(W515L) [76%]	YES
	#27	NGS	c.1543_1544delinsAA; p.(W515K) [40%]	c.1543_1544delinsAA; p.(W515K) [10%]	YES
*MPL* exon 10	#28	NGS	c.1544G>T; p.(W515L) [39%]	c.1544G>T; p.(W515L) [26%]	YES
	#29	NGS	c.1543_1544delinsAA; p.(W515K) [42%]	c.1543_1544delinsAA; p.(W515K) [27%]	YES
	#30	Sanger	Non-mutated	Non-mutated	YES
	#31	Sanger	Non-mutated	Non-mutated	YES
	#32	Sanger	Non-mutated	Non-mutated	YES
	#33	Sanger	Non-mutated	Non-mutated	YES
	#34	Sanger	Non-mutated	Non-mutated	YES
*CSF3R* exon 17	#35	NGS	c.2503G>A; p.(E835K) [50%]	c.2503G>A; p.(E835K) [47%]	YES
	#36	NGS	c.2503G>A; p.(E835K) [50%]	c.2503G>A; p.(E835K) [46%]	YES
	#37	NGS	c.2503G>A; p.(E835K) [50%]	c.2503G>A; p.(E835K) [47%]	YES
*CSF3R* exon 14	#38	NGS	c.1853C>T; p.(T618I) [38%]	c.1853C>T; p.(T618I) [30%]	YES
*CSF3R* exon 17	#38	NGS	c.2326C>T; p.(Q776*) [84%]	c.2326C>T; p.(Q776*) [70%]	YES
*CSF3R* exon 17	#39	NGS	c.2386C>T; p.(R796C) [39%]	c.2386C>T; p.(R796C) [57%]	YES
*CSF3R* exon 14	#40	NGS	c.1853C>T; p.(T618I) [49%]	c.1853C>T; p.(T618I) [32%]	YES
*CSF3R* exon 17	#41	NGS	c.2503G>A; p.(E835K) [50%]	c.2503G>A; p.(E835K) [47%]	YES
*CSF3R* exons 14 and 17	#42	Sanger	Non-mutated	Non-mutated	YES
	#43	Sanger	Non-mutated	Non-mutated	YES
	#44	Sanger	Non-mutated	Non-mutated	YES
	#45	Sanger	Non-mutated	Non-mutated	YES
	#46	Sanger	Non-mutated	Non-mutated	YES
*SETBP1* exon 4	#47	NGS	c.2602G>T; p.(D868Y) [44%]	c.2602G>T; p.(D868Y) [41%]	YES
	#48	Sanger	c.2602G>T; p.(D868Y)	c.2602G>T; p.(D868Y) [40%]	YES
	#49	NGS	c.2602G>A; p.(D868N) [51%]	c.2602G>A; p.(D868N) [47%]	YES
	#50	NGS	c.2608G>A; p.(G870S) [46%]	c.2608G>A; p.(G870S) [55%]	YES
	#51	Sanger	c.2608G>A; p.(G870S)	c.2608G>A; p.(G870S) [50%]	YES
	#52	Sanger	c.2612T>C; p.(I871T)	c.2612T>C; p.(I871T) [52%]	YES
	#53	Sanger	c.2602G>A; p.(D868N)	c.2602G>A; p.(D868N) [45%]	YES
	#54	Sanger	Non-mutated	Non-mutated	YES
	#55	Sanger	Non-mutated	Non-mutated	YES
	#56	Sanger	Non-mutated	Non-mutated	YES
	#57	Sanger	Non-mutated	Non-mutated	YES
	#58	Sanger	Non-mutated	Non-mutated	YES

**Table 3:** Comparison of variants detected by the Sanger/NGS method vs. MinION. The genes included in this table have clinical significance in the diagnosis, prognosis, or development of MDS.

Gene	Sample ID	Gold Standard Method	Sanger/NGS Detected Variants [VAF Value]	MinION Detected Variants [VAF Value]	Matching Results
*SF3B1* exon 15	#59	Sanger	c.2098A>G; p.(K700E)	c.2098A>G; p.(K700E) [30%]	YES
	#60	Sanger	c.2098A>G; p.(K700E)	c.2098A>G; p.(K700E) [36%]	YES
	#61	Sanger	c.2098A>G; p.(K700E)	c.2098A>G; p.(K700E) [36%]	YES
*SF3B1* exon 14	#62	Sanger	c.1997A>G; p.(K666R)	c.1997A>G; p.(K666R) [57%]	YES
*SF3B1* exon 15	#63	Sanger	c.2098A>G; p.(K700E)	c.2098A>G; p.(K700E) [35%]	YES
*SF3B1* exons 12–16	#64	Sanger	Non-mutated	Non-mutated	YES
	#65	Sanger	Non-mutated	Non-mutated	YES
	#66	Sanger	Non-mutated	Non-mutated	YES
	#67	Sanger	Non-mutated	Non-mutated	YES
	#68	Sanger	Non-mutated	Non-mutated	YES

**Table 4:** Comparison of variants detected by the Sanger/NGS method vs. MinION. The genes included in this table have clinical significance in the diagnosis, prognosis, or development of AML.

Gene	Sample ID	Gold Standard Method	Sanger/NGS Detected Variants [VAF Value]	MinION Detected Variants [VAF Value]	Matching Results
*NPM1* exon 12	#69	Sanger	Type A: c.863_864insTCTG; p.(W288Cfs*12)	c.863_864insTCTG; p.(W288Cfs*12) [33%]	YES
	#70	Sanger	Type A: c.863_864insTCTG; p.(W288Cfs*12)	c.863_864insTCTG; p.(W288Cfs*12) [34%]	YES
	#71	Sanger	Type A: c.863_864insTCTG; p.(W288Cfs*12)	c.863_864insTCTG; p.(W288Cfs*12) [22%]	YES
	#72	Sanger	Type B: c.863_864insCATG; p.(W288Cfs*12)	c.863_864insCATG; p.(W288Cfs*12) [41%]	YES
	#73	Sanger	Type B: c.863_864insCATG; p.(W288Cfs*12)	c.863_864insCATG; p.(W288Cfs*12) [45%]	YES
	#74	NGS	Type D: c.863_864insCCTG; p.(W288Cfs*12) [42%]	c.863_864insCCTG; p.(W288Cfs*12) [42%]	YES
	#75	NGS	Type D: c.863_864insCCTG; p.(W288Cfs*12) [44%]	c.863_864insCCTG; p.(W288Cfs*12) [38%]	YES
	#76	Sanger	Type D: c.863_864insCCTG; p.(W288Cfs*12)	c.863_864insCCTG; p.(W288Cfs*12) [38%]	YES
	#77	Sanger	Type D: c.863_864insCCTG; p.(W288Cfs*12)	c.863_864insCCTG; p.(W288Cfs*12) [38%]	YES
	#78	Sanger	Type D: c.863_864insCCTG; p.(W288Cfs*12)	c.863_864insCCTG; p.(W288Cfs*12) [32%]	YES
	#79	Sanger	Non-mutated	Non-mutated	YES
	#80	Sanger	Non-mutated	Non-mutated	YES
*KIT* exon 17	#81	NGS	c.2447A>T; p.(D816V) [16%]	c.2447A>T; p.(D816V) [16%]	YES
	#82	NGS	c.2447A>T; p.(D816V) [14%]	c.2447A>T; p.(D816V) [14%]	YES
	#83	NGS	c.2447A>T; p.(D816V) [26%]	c.2447A>T; p.(D816V) [27%]	YES
*KIT* exons 8 and 17	#84	Sanger	Non-mutated	Non-mutated	YES
	#85	Sanger	Non-mutated	Non-mutated	YES
	#86	Sanger	Non-mutated	Non-mutated	YES
	#87	Sanger	Non-mutated	Non-mutated	YES
	#88	Sanger	Non-mutated	Non-mutated	YES
*IDH2* exon 4	#89	NGS	c.419G>A; p.(R140Q) [44%]	c.419G>A; p.(R140Q) [54%]	YES
*IDH1* exon 4	#90	NGS	c.394C>G; p.(R132G) [5%]	c.394C>G; p.(R132G) [8%]	YES
	#91	Sanger	c.394C>G; p.(R132G)	c.394C>G; p.(R132G) [64%]	YES
	#92	NGS	c.395G>A; p.(R132H) [43%]	c.395G>A; p.(R132H) [50%]	YES
*IDH1* exon 4	#93	NGS	c.395G>A; p.(R132H) [22%]	c.395G>A; p.(R132H) [46%]	YES
*IDH2* exon 4	#94	NGS	c.419G>A; p.(R140Q) [41%]	c.419G>A; p.(R140Q) [58%]	YES
	#95	NGS	c.419G>A; p.(R140Q) [45%]	c.419G>A; p.(R140Q) [59%]	YES
*IDH1* exon 4 and *IDH2* exon 4	#96	Sanger	Non-mutated	Non-mutated	YES
	#97	Sanger	Non-mutated	Non-mutated	YES
	#98	Sanger	Non-mutated	Non-mutated	YES
	#99	Sanger	Non-mutated	Non-mutated	YES
	#100	Sanger	Non-mutated	Non-mutated	YES
*CEBPA* exon 1	#101	Sanger	c.303_304delinsT; p.(G102Afs*58)	c.303_304delinsT; p.(G102Afs*58) [51%]	YES
	#101	Sanger	c.962A>G; p.(N321S)	c.962A>G; p.(N321S) [53%]	YES
	#102	Sanger	c.570_571insGCACCC; p.(S190_H191insAP)	c.570_571insGCACCC; p.(S190_H191insAP) [22%]	YES
	#103	Sanger	c.213_216delCGCC; p.(A72Sfs*87)	c.213_216delCGCC; p.(A72Sfs*87) [19%]	YES
	#104	Sanger	c.482A>T; p.(K161M)	Non-mutated	NO*
	#105	Sanger	c.146C>T; p.(P49L)	c.146C>T; p.(P49L) [55%]	YES
	#106	Sanger	c.570_571insGCACCC; p.(S190_H191insAP)	c.570_571insGCACCC; p.(S190_H191insAP) [25%]	YES
	#107	Sanger	Non-mutated	Non-mutated	YES
	#108	Sanger	Non-mutated	Non-mutated	YES
	#109	Sanger	Non-mutated	Non-mutated	YES
	#110	Sanger	Non-mutated	Non-mutated	YES
	#111	Sanger	Non-mutated	Non-mutated	YES
*NRAS* exon 3	#112	NGS	c.183A>T; p.(Q61H) [35%]	c.183A>T; p.(Q61H) [48%]	YES
*NRAS* exon 2	#113	NGS	c.34G>A; p.(G12S) [17%]	c.34G>A; p.(G12S) [16%]	YES
	#113	NGS	c.35G>A; p.(G12D) [10%]	c.35G>A; p.(G12D) [11%]	YES
	#114	NGS	c.35G>T; p.(G12V) [17%]	c.35G>T; p.(G12V) [15%]	YES
*NRAS* exon 3	#115	NGS	c.182A>G; p.(Q61R) [43%]	c.182A>G; p.(Q61R) [32%]	YES
*NRAS* exon 2	#116	NGS	c.34G>A; p.(G12S) [24%]	c.34G>A; p.(G12S) [22%]	YES
*NRAS* exon 2–3	#117	Sanger	Non-mutated	Non-mutated	YES
	#118	Sanger	Non-mutated	Non-mutated	YES
	#119	Sanger	Non-mutated	Non-mutated	YES
	#120	Sanger	Non-mutated	Non-mutated	YES
	#121	Sanger	Non-mutated	Non-mutated	YES
*KRAS* exon 2	#122	NGS	c.35G>C; p.(G12A) [7%]	c.35G>C; p.(G12A) [7%]	YES
	#122	NGS	c.34G>A; p.(G12S) [5%]	c.34G>A; p.(G12S) [6%]	YES
	#123	NGS	c.35G>C; p.(G12A) [38%]	c.35G>C; p.(G12A) [35%]	YES
	#124	NGS	c.35G>C; p.(G12A) [9%]	c.35G>C; p.(G12A) [9%]	YES
	#125	NGS	c.35G>C; p.(G12A) [38%]	c.35G>C; p.(G12A) [38%]	YES
	#126	NGS	c.34G>C; p.(G12R) [14%]	c.34G>C; p.(G12R) [10%]	YES
	#127	NGS	c.35G>A; p.(G12D) [47%]	c.35G>A; p.(G12D) [47%]	YES
*KRAS* exon 2–3	#128	Sanger	Non-mutated	Non-mutated	YES
	#129	Sanger	Non-mutated	Non-mutated	YES
	#130	Sanger	Non-mutated	Non-mutated	YES
	#131	Sanger	Non-mutated	Non-mutated	YES
	#132	Sanger	Non-mutated	Non-mutated	YES
*TP53* exons 5–9	#133	NGS	c.743G>A; p.(R248Q) [21%]	c.743G>A; p.(R248Q) [30%]	YES
*TP53* exons 5–6	#134	NGS	c.216del; p.(V73Wfs*50) [98%]	c.216del; p.(V73Wfs*50) [88%]	YES
*TP53* exons 5–6	#135	Sanger	c.488A>G; p.(Y163C)	c.488A>G; p.(Y163C) [89%]	YES
*TP53* exons 8–9	#136	Sanger	c.833C>T; p.(P278L)	c.833C>T; p.(P278L) [13%]	YES
*TP53* exons 5–6	#137	Sanger	c.535C>T; p.(H179Y)	c.535C>T; p.(H179Y) [81%]	YES
	#138	Sanger	c.641A>G; p.(H214R)	c.641A>G; p.(H214R) [39%]	YES
*TP53* exons 8–9	#139	Sanger	c.853G>A; p.(E285K)	c.853G>A; p.(E285K) [88%]	YES
*TP53* exons 3–10	#140	Sanger	Non-mutated	Non-mutated	YES
	#141	Sanger	Non-mutated	Non-mutated	YES
	#142	Sanger	Non-mutated	Non-mutated	YES
	#143	Sanger	Non-mutated	Non-mutated	YES
	#144	Sanger	Non-mutated	Non-mutated	YES

* Unique variant in all validations in which there was no concordance with MinION sequencing results.

**Table 5:** Comparison of variants detected by the Sanger/NGS method vs. MinION. The genes included in this table have clinical significance in the diagnosis, prognosis, or development of CML.

Gene	Sample ID	Gold Standard Method	Sanger Detected Variants	MinION Detected Variants [VAF Value]	Matching Results
*ABL1* exon 4	#145	Sanger	c.763G>A; p.(E255K)	c.763G>A; p.(E255K) [94%]	YES
*ABL1* exon 6	#146	Sanger	c.944C>T; p.(T315I)	c.944C>T; p.(T315I) [99%]	YES
*ABL1* exon 4	#147	Sanger	c.749G>A; p.(G250E)	c.749G>A; p.(G250E) [99%]	YES
	#148	Sanger	c.730A>G; p.(M244V)	c.730A>G; p.(M244V) [8%]	YES
	#149	Sanger	c.749G>A; p.(G250E)	c.749G>A; p.(G250E) [38%]	YES
	#150	Sanger	c.749G>A; p.(G250E)	c.749G>A; p.(G250E) [34%]	YES
*ABL1* exons 4–7	#151	Sanger	Non-mutated	Non-mutated	YES
*ABL1* exons 4–7	#152	Sanger	Non-mutated	Non-mutated	YES
*ABL1* exons 4–7	#153	Sanger	Non-mutated	Non-mutated	YES
*ABL1* exons 4–7	#154	Sanger	Non-mutated	Non-mutated	YES

**Table 6:** Sensitivity test to check the high detection sensitivity of the MinION technology in variants with VAFs < 5%.

Gene: Disease	Sample ID	Gold Standard Method	NGS Detected Variants [VAF]	MinION Detected Variants [VAF]	Matching Results
*SF3B1* exon 14: *MDS*	#155	NGS	c.1997A>C; p.(K666T) [1%]	c.1997A>C; p.(K666T) [2%]	YES
	#156	NGS	c.1874G>T; p.(R625L) [1%]	c.1874G>T; p.(R625L) [10%]	YES
*KIT* exon 17: *AML*	#157	NGS	c.2447A>T; p.(D816V) [2%]	c.2447A>T; p.(D816V) [2%]	YES
	#158	NGS	c.2446G>C; p.(D816H) [4%]	c.2446G>C; p.(D816H) [6%]	YES
	#159	NGS	c.2466T>G; p.(N822K) [2%]	c.2466T>G; p.(N822K) [2%]	YES
	#160	NGS	c.2447A>T; p.(D816V) [1%]	c.2447A>T; p.(D816V) [1%]	YES
*IDH1* exon *4: AML*	#93	NGS	c.394C>G; p.(R132G) [2%]	c.394C>G; p.(R132G) [1%]	YES
*IDH2* exon *4: AML*	#93	NGS	c.419G>A; p.(R140Q) [2%]	c.419G>A; p.(R140Q) [6%]	YES
*NRAS* exon 2: *AML*	#112	NGS	c.38G>A; p.(G13D) [1%]	c.38G>A; p.(G13D) [2%]	YES
	#161	NGS	c.38G>A; p.(G13D) [3%]	c.38G>A; p.(G13D) [4%]	YES
	#161	NGS	c.38G>T; p.(G13V) [3%]	c.38G>T; p.(G13V) [2%]	YES
	#161	NGS	c.34G>A; p.(G12S) [2%]	c.34G>A; p.(G12S) [1%]	YES
	#162	NGS	c.38G>A; p.(G13D) [3%]	c.38G>A; p.(G13D) [5%]	YES
*KRAS* exon 2: *AML*	#163	NGS	c.38G>A; p.(G13D) [1%]	c.38G>A; p.(G13D) [2%]	YES
	#122	NGS	c.34G>T; p.(G12C) [4%]	c.34G>T; p.(G12C) [4%]	YES
	#164	NGS	c.38G>A; p.(G13D) [2%]	c.38G>A; p.(G13D) [3%]	YES

## Data Availability

The data presented in this study are available on request from the corresponding author.

## Supplementary Materials

The following supporting information can be downloaded at: https://www.mdpi.com/article/10.3390/cancers17111811/s1, Table S1: Samples information and organization along the manuscript. Main information about the samples is presented in the following table. Moreover, the tables in the main text in which these samples are analyzed are specified. Samples #1 to #58 correspond to myeloproliferative neoplasms (MPN). Samples #59 to #68 (plus samples #155 and #156) correspond to myelodysplastic syndromes (MDS). Samples #69 to #144 and #157 to #164, correspond to acute myeloid leukemia (AML). Samples #145 to #153 correspond to chronic myeloid leukemia (CML). Finnaly, sample #154 correspond to acute lymphoblastic leukemia (ALL). Table S2: Primers and conditions used for PCRs. The sequence of primers used was specified for each gene. All primers are based on the hg19 version of the human reference genome.
