# Supplementary material for: From Sanger to Oxford Nanopore MinION Technology: The Impact of Third-Generation Sequencing on Genetic Hematological Diagnosis
Source: Cancers (Basel). 2025 May 29;17(11):1811. doi: 10.3390/cancers17111811 (PMC12153771; doi:10.3390/cancers17111811)
Supplement: Supplementary file 1 [file cancers-17-01811-s001.zip › cancers-3598191-supplementary.pdf]

Supplementary Tables

Table S1: Samples information and organization along the manuscript. Main information about the samples is presented in the following table. Moreover, the tables in the main text in which these samples are analyzed are specified. Samples #1 to #58 correspond to myeloproliferative neoplasms (MPN). Samples #59 to #68 (plus samples #155 and #156) correspond to myelodysplastic syndromes (MDS). Samples #69 to #144 and #157 to #164, correspond to acute myeloid leukemia (AML). Samples #145 to #153 correspond to chronic myeloid leukemia (CML). Finnaly, sample #154 correspond to acute lymphoblastic leukemia (ALL).

Sample ID	Disease	Nº of regions of interest (ROI) analyzed	Sample results location (Nº of table(s) in the main text)
#1	MPN	1	Table 2
#2	MPN	1	Table 2
#3	MPN	1	Table 2
#4	MPN	1	Table 2
#5	MPN	1	Table 2
#6	MPN	1	Table 2
#7	MPN	1	Table 2
#8	MPN	1	Table 2
#9	MPN	1	Table 2
#10	MPN	1	Table 2
#11	MPN	1	Table 2
#12	MPN	1	Table 2
#13	MPN	1	Table 2
#14	MPN	1	Table 2
#15	MPN	1	Table 2
#16	MPN	1	Table 2
#17	MPN	1	Table 2
#18	MPN	1	Table 2
#19	MPN	1	Table 2
#20	MPN	1	Table 2
#21	MPN	1	Table 2
#22	MPN	1	Table 2
#23	MPN	1	Table 2
#24	MPN	1	Table 2
#25	MPN	1	Table 2
#26	MPN	1	Table 2
#27	MPN	1	Table 2
#28	MPN	1	Table 2
#29	MPN	1	Table 2
#30	MPN	1	Table 2
#31	MPN	1	Table 2
#32	MPN	1	Table 2
#33	MPN	1	Table 2
#34	MPN	1	Table 2
#35	MPN	1	Table 2
#36	MPN	1	Table 2
#37	MPN	1	Table 2
#38	MPN	2	Table 2 (2 ROI)
#39	MPN	1	Table 2

#40	MPN	1	Table 2
#41	MPN	1	Table 2
#42	MPN	1	Table 2
#43	MPN	1	Table 2
#44	MPN	1	Table 2
#45	MPN	1	Table 2
#46	MPN	1	Table 2
#47	MPN	1	Table 2
#48	MPN	1	Table 2
#49	MPN	1	Table 2
#50	MPN	1	Table 2
#51	MPN	1	Table 2
#52	MPN	1	Table 2
#53	MPN	1	Table 2
#54	MPN	1	Table 2
#55	MPN	1	Table 2
#56	MPN	1	Table 2
#57	MPN	1	Table 2
#58	MPN	1	Table 2
#59	MDS	1	Table 3
#60	MDS	1	Table 3
#61	MDS	1	Table 3
#62	MDS	1	Table 3
#63	MDS	1	Table 3
#64	MDS	1	Table 3
#65	MDS	1	Table 3
#66	MDS	1	Table 3
#67	MDS	1	Table 3
#68	MDS	1	Table 3
#69	AML	1	Table 4
#70	AML	1	Table 4
#71	AML	1	Table 4
#72	AML	1	Table 4
#73	AML	1	Table 4
#74	AML	1	Table 4
#75	AML	1	Table 4
#76	AML	1	Table 4
#77	AML	1	Table 4
#78	AML	1	Table 4
#79	AML	1	Table 4
#80	AML	1	Table 4
#81	AML	1	Table 4
#82	AML	1	Table 4
#83	AML	1	Table 4
#84	AML	1	Table 4
#85	AML	1	Table 4
#86	AML	1	Table 4
#87	AML	1	Table 4
#88	AML	1	Table 4
#89	AML	1	Table 4
#90	AML	1	Table 4

#91	AML	1	Table 4
#92	AML	1	Table 4
#93	AML	3	Table 4 (1 ROI) and Table 6 (2 ROI)
#94	AML	1	Table 4
#95	AML	1	Table 4
#96	AML	1	Table 4
#97	AML	1	Table 4
#98	AML	1	Table 4
#99	AML	1	Table 4
#100	AML	1	Table 4
#101	AML	2	Table 4 (2 ROI)
#102	AML	1	Table 4
#103	AML	1	Table 4
#104	AML	1	Table 4
#105	AML	1	Table 4
#106	AML	1	Table 4
#107	AML	1	Table 4
#108	AML	1	Table 4
#109	AML	1	Table 4
#110	AML	1	Table 4
#111	AML	1	Table 4
#112	AML	2	Table 4 (1 ROI) and Table 6 (1 ROI)
#113	AML	2	Table 4 (2 ROI)
#114	AML	1	Table 4
#115	AML	1	Table 4
#116	AML	1	Table 4
#117	AML	1	Table 4
#118	AML	1	Table 4
#119	AML	1	Table 4
#120	AML	1	Table 4
#121	AML	1	Table 4
#122	AML	3	Table 4 (2 ROI) and Table 6 (1 ROI)
#123	AML	1	Table 4
#124	AML	1	Table 4
#125	AML	1	Table 4
#126	AML	1	Table 4
#127	AML	1	Table 4
#128	AML	1	Table 4
#129	AML	1	Table 4
#130	AML	1	Table 4
#131	AML	1	Table 4
#132	AML	1	Table 4
#133	AML	1	Table 4
#134	AML	1	Table 4
#135	AML	1	Table 4
#136	AML	1	Table 4
#137	AML	1	Table 4
#138	AML	1	Table 4
#139	AML	1	Table 4
#140	AML	1	Table 4
#141	AML	1	Table 4

#142	AML	1	Table 4
#143	AML	1	Table 4
#144	AML	1	Table 4
#145	CML	1	Table 5
#146	CML	1	Table 5
#147	CML	1	Table 5
#148	CML	1	Table 5
#149	CML	1	Table 5
#150	CML	1	Table 5
#151	CML	1	Table 5
#152	CML	1	Table 5
#153	CML	1	Table 5
#154	ALL	1	Table 5
#155	MDS	1	Table 6
#156	MDS	1	Table 6
#157	AML	1	Table 6
#158	AML	1	Table 6
#159	AML	1	Table 6
#160	AML	1	Table 6
#161	AML	3	Table 6 (3 ROI)
#162	AML	1	Table 6
#163	AML	1	Table 6
#164	AML	1	Table 6

Supplementary Table S2

Table S2: **Primers and conditions used for PCRs.** The sequence of primers used was specified for each gene. All primers are based on the hg19 version of the human reference genome.

Gene	Exon	RefSeq	Forward primer (5´-3´)	Reverse primer (5´-3´)	Hybridization temperature
CALR	9	NM_004343.4	CCTGCAGGCAGCAGAGAAAC	ACAGAGACATTATTTGGCGCG	55ºC
JAK2	12	NM_004972.4	CTCCTCTTTGGAGCAATTCA	GAGAACTTGGGAGTTGCGATA	55ºC
	14		TCCTCAGAACGTTGATGGCAG	ATTGCTTTCCTTTTTCACAAGAT	59ºC
MPL	10	NM_005373.3	TGGGCCGAAGTCTGACCCTTT	ACAGAGCGAACCAAGAATGCCTG T	59ºC
SETBP1	3	NM_015559.3	CCACTTTCAACACAGTTAGGTG	TCTCGTGGTAGAAGGTGTAACTC	55ºC
CSF3R	14	NM_000760.4	CCACGGAGGCAGCTTTAC	AAATCAGCATCCTTTGGGTG	56ºC
	17		CTGTCACTTCCGGCAACAT	TGGCCCAAAGACACAGTCGT	58ºC
NPM1	12	NM_002520.7	TTAACTCTCTGGTGGTAGAATGAA	CAAGACTATTTGCCATCCTAAC	55ºC
TP53	3-4	NM_000546.6	GTGGGAAGCGAAAATTCCAT	GCCAGGCATTGAAGTCTCAT	59ºC
	5-6		TGTTCACTTGTGCCCTGACT	TTAACCCCTCCTCCCAGAGA	59ºC
	7		GAGCTTGCAGTGAGCTGAGA	GGGATGTGATGAGAGGTGGA	60ºC
	8-9		GACAAGGGTGGTTGGGAGTA	GCCCCAATTGCAGGTAAAAC	59ºC
	10		TGCATGTTGCTTTTGTACCGT	TCAGCTGCCTTTGACCATGA	59ºC
KIT	8	NM_000222.3	GCTGAGGTTTTCCAGCACTC	AATTGCAGTCCTTCCCCTCT	58ºC
	17		TTCACTCTTTACAAGTTAAAATG	GGACTGTCAAGCAGAGAATG	60ºC
IDH1	4	NM_005896.4	TGCCACCAACGACCAAGTCA	TGTGTTGAGATGGACGCCTATTTG	56ºC
IDH2	4	NM_002168.4	GGGGTTCAAATTCTGGTTGA	CTAGGCGAGGAGCTCCAGT	58ºC
CEBPA ¶	1	NM_004364.5	CGCCATGCCGGGAGAACTCT	GCCTTGGCCTTCTCCTGCTG	65ºC
			GACCTGTTCCAGCACAGCCG	CCCGGTACTCGTTGCTGTTCTTGT C	61ºC
			GGGCAAGGCCAAGAAGTCGG	CCTCACGCGCAGTTGCCCAT	65ºC
ABL1 §	4, 6 y 8	NM_005157.6	P190 GAACTCGCAACAGTCCTTCGAC	P190 CGGACTTGATGGAGAACTTG	p190 and p210 58ºC (1st PCR) 70ºC (2nd PCR)
			P210 GAAGCTTCTCCCTGACATCCGT	P210 CGGACTTGATGGAGAACTTG	

¶Exon 1 of the *CEBPA* gene is divided into three fragments, each amplified by a corresponding primer pair, encompassing key mutation hotspots of highest diagnostic relevance.

§Amplification of the *ABL1* gene required a nested PCR approach, thereby requiring the use of four primers. This amplification targeted the kinase domain region of the *BCR::ABL1* fusion gene.
